# Effectiveness of Proton-Pump Inhibitors in Chronic Obstructive Pulmonary Disease: A Meta-Analysis of Randomized Controlled Trials

**DOI:** 10.3389/fmed.2022.841155

**Published:** 2022-02-16

**Authors:** Fei Yu, Qihui Huang, Yousheng Ye, Lin Zhang

**Affiliations:** The First People's Hospital of Hefei, Hefei, China

**Keywords:** proton pump inhibitors, chronic obstructive pulmonary disease, clinical efficacy, gastroesophageal reflux disease, meta-analysis

## Abstract

**Background:**

Although several randomized controlled trials (RCTs) have been published in recent years, the role of proton-pump inhibitors (PPI) in patients with chronic obstructive pulmonary disease (COPD) remains controversial. This preliminary meta-analysis was conducted to evaluate the clinical efficacy of PPI in patients with COPD.

**Methods:**

RCTs related to PPI in the treatment of patients with a definite diagnosis of COPD were enrolled in this meta-analysis. PubMed, Embase, Cochrane Library, CNKI, Wanfang and VIP databases were retrieved to identify eligible studies from database establishment to September 22, 2021. Two researchers independently screened the articles, extracted the data and evaluated the risk of bias in the included studies independently. The study complied with PRISMA 2020 guideline for this study. The meta-analysis was performed using RevMan 5.3. Heterogeneity among studies was tested using the *I*^2^ test. The results were presented as risk ratios (RRs) with 95% confidence intervals (CIs).

**Results:**

A total of 15 RCTs, including 1,684 patients, were enrolled. The meta-analysis revealed that PPI plus conventional treatment was superior to conventional treatment with respect to the case fatality rate (RR = 0.30; 95% CI, 0.18–0.52; *P* < 0.001), the incidence of gastrointestinal bleeding (RR = 0.23; 95% CI, 0.14–0.38; *P* < 0.001), the incidence of other adverse reactions (RR = 0.33; 95% CI, 0.28–0.39; *P* < 0.001) and the number of acute exacerbations [mean difference (MD) = −1.17; 95% CI, 1.75 to −0.60: *P* < 0.001] in patients with COPD. No significant differences were found in clinical efficacy (RR = 1.08; 95% CI, 0.95–1.22; *P* = 0.25), FEV1/FVC (MD = 3.94; 95% CI, −8.70 to 16.58; *P* = 0.54) and nosocomial infection rate (RR = 1.31; 95% CI, 0.57–3.00; *P* = 0.52) between the two groups.

**Discussion:**

This comprehensive meta-analysis suggested that PPI treatment for COPD may reduce the case fatality rate, incidence of gastrointestinal bleeding and other adverse reactions and number of acute exacerbations. However, the present meta-analysis also has some limitations of the evidence, such as the high risk of bias of the included studies, and predominance of included studies from China, which may result in publication bias. Therefore, further large-scale RCTs are needed to confirm our findings.

**Systematic Trial Registration:**

Identifier: CRD42022301304.

## Introduction

Chronic obstructive pulmonary disease (COPD) is a respiratory disease characterized by persistent airflow limitation and dyspnoea. It is the third leading cause of death worldwide and causes a huge economic burden to society because of its chronic disease course, repeated acute exacerbations and effects on performing activities of daily living (1). Previous studies have shown that the prevalence of gastroesophageal reflux disease (GERD) in patients with COPD was higher than that in the normal population. GERD may cause acute exacerbation of COPD and is considered an independent risk factor for COPD death (2–4). COPD and GERD are mutually causal, forming a vicious circle, which seriously affects the quality of life (5). Proton-pump inhibitors (PPI) are the first-line drugs for the treatment of GERD. PPI therapy for patients with COPD complicated with GERD may reduce the number of acute exacerbations of COPD, thus delaying disease progression of the disease and improving clinical outcomes.

Thus, far, the pathogenesis of COPD remains unclear. Many patients with COPD complicated with GERD have not received formal diagnosis and treatment, and numerous GERD cases are asymptomatic. In addition, the systemic efficacy and mortality risk of PPI in patients with COPD are controversial. Herein, we conducted a comprehensive meta-analysis of randomized controlled trials (RCTs) to explore the clinical efficacy and safety of PPI therapy in patients with COPD.

## Methods

### Eligibility Criteria

RCTs were strictly screened following the PICOS principle (participants, interventions, comparisons, outcomes and study design). The inclusion criteria were as follows: (1) the participants were diagnosed with COPD according to the COPD Global Initiative (GOLD guidelines) (6); (2) RCTs compared conventional treatment plus PPI treatment with conventional treatment alone, and (3) RCTs were included regardless of the absence or presence of blind. The exclusion criteria were as follows: (1) basic experiments, animal experiments, repeated publications, and documents that cannot extract key information such as intervention measures and outcome indicators, and (2) the articles that have obvious experimental design errors or data errors.

### Information Sources and Search Strategy

The search conducted using a combination of subject terms and entry terms as follows: Pulmonary Disease, Chronic Obstructive, Chronic Obstructive Lung Disease, Chronic Obstructive Pulmonary Diseases, COAD, COPD, Chronic Obstructive Airway Disease, Chronic Obstructive Pulmonary Disease, Airflow Obstruction, Chronic, Airflow Obstructions, Chronic, Chronic Airflow Obstructions, Chronic Airflow Obstruction, Proton-pump Inhibitors, Inhibitors, Proton-pump, Proton-pump Inhibitor, Inhibitor, Proton-pump, Pump Inhibitor, Proton, Omeprazole, Esomeprazole, Esomeprazole, Rabeprazole, Pantoprazole, Ilaprazole, Lansoprazole, etc. Moreover, the references of the included articles were screened to determine appropriate related studies.

### Data Collection and Quality Assessment

Two researchers (Fei Yu and Qi-hui Huang) screened the articles and extracted data independently according to the PRISMA 2020 recommendations (7). Discussion or third-party negotiation was conducted in case of a dispute. If necessary, the authors of the enrolled studies were contacted by email or telephone to obtain information that was not presented in the article but was important to the present study. The following details were extracted from each study: basic information of the studies, baseline characteristics of the research object, test grouping, specific treatment measures, key elements of the bias risk assessment, outcome indicators and specific data.

In this study, the primary outcome indicators were the case fatality rate and clinical efficacy. The secondary outcome indicators include forced expiratory volume in 1/forced vital capacity (FEV1/FVC), gastrointestinal bleeding, other adverse reactions, nosocomial infections and number of acute exacerbations. Two investigators independently (Fei Yu and Qi-hui Huang) evaluated the risk of bias in the included studies and cross-checked the results. The second version of the Cochrane tool for assessing RoB in RCTs (RoB2) was used (8). This tool consists of the following five parts: bias arising from the randomization process, bias due to deviations from intended interventions, bias due to missing outcome data, bias in the measurement of the outcomes and bias in the selection of the reported result. The risk levels are classified as low risk of bias, some concerns and high risk of bias. In addition, the Grading of Recommendations, Assessment, Development and Evaluations (GRADE) was used to rate the level of evidence of the outcomes obtained in this study (9). Assessment of the quality of evidence considers five aspects: limitations, inconsistencies, indirectness, inaccuracy and publication bias.

### Statistical Analyses

RevMan 5.3 was used for statistical analysis. Results of the meta-analysis of categorical variables were presented as Risk Ratios (RRs) with 95% confidence intervals (CIs), and the results of the analysis of continuous variables were presented as mean difference (MD) with 95% CIs. The results were expressed by *P*-value, and *P* < 0.05 indicated the difference between two intervention measures. Heterogeneity test was conducted on the results of the study. The *I*^2^ test was used to analyze the heterogeneity among the results of the study within the group. If the heterogeneity test results were *P* ≥ 0.1 and *I*^2^ < 50%, the fixed-effects model was selected. In contrast, if *P* < 0.1 or *I*^2^ ≥ 50%, the random-effects model was used. Sensitivity analysis and subgroup analysis were conducted when the heterogeneity was significant.

## Results

**Figure 1:**
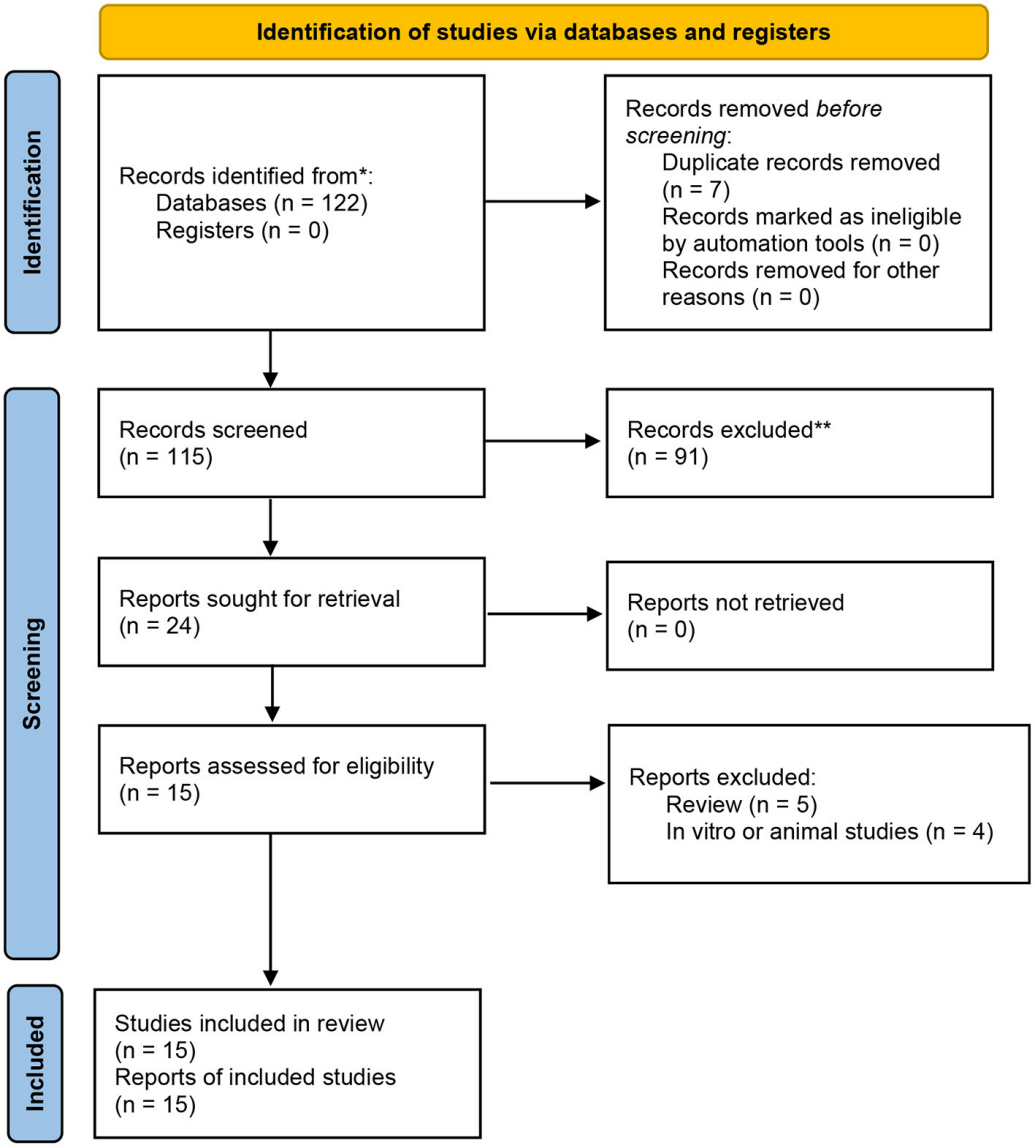
The PRISMA 2020 flow chart.

In the present meta-analysis, 122 relevant studies were retrieved: 9 from PubMed, 39 from EMBASE, 1 from the Cochrane Library, 21 from the VIP, 33 from the CNKI and 28 from the WanFang database. After screening, 15 RCTs (10–24) were identified for this meta-analysis. Overall, these studies included 1,684 patients, 806 of whom received conventional treatment (control group) and 878 of whom received conventional treatment pklus PPI therapy (PPI group). The details of literature search and selection procedures are showed in Figure 1. The basic information of all patients is shown in Table 1.

**Table 1:** The basic characteristics of involved trials.

**References**	**Study period**	**Patients**	**Sample size**	**Mean medical history**	**Intervention**		**Outcome indicators**
			**T/C**	**T/C**	**T**	**C**	
Sasaki et al. (10)	2005.10–2007.03	COPD	50/50	/	CT + Lansoprazole 15 mg QD	CT	g
Huang (11)	2016.11–2018.02	COPD + AE + RF	40/40	/	CT + Pantoprazole 40 mg Q12H	CT	ade
Wang (12)	2013.04–2014.10	COPD + AE + RF	100/100	5.78 ± 2.84/ 5.58 ± 2.92	CT + Pantoprazole 40 mg Q12H	CT	ade
Li (13)	2015.10–2016.10	COPD + AE + RF	31/31	5.43 ± 2.34/ 5.76 ± 2.15	CT + Pantoprazole 40 mg Q12H	CT	ade
Xiong (14)	2016.10–2017.10	COPD + AE + RF	32/32	4.5 ± 3.3/ 4.0 ± 3.0	CT + Pantoprazole 40 mg Q12H	CT	bde
Zhen (15)	2014.03–2016.04	COPD + AE + RF	34/34	10.2 ± 1.3/ 9.8 ± 1.4	CT + Pantoprazole 40 mg BID	CT	abe
Gu (16)	2016.01–2017.09	COPD + RF	32/35	15.6 ± 2.4/ 15.9 ± 2.6	CT + Pantoprazole 40 mg QD	CT	ade
Xu and Jiao (17)	2013.01–2014.03	COPD + AE + RF	50/50	/	CT + Pantoprazole 40 mg BID	CT	ade
Hu (18)	2013.07–2014.08	COPD	63/63	4.89 ± 1.33	CT + Omeprazole 20 mg QD	CT	defg
Hu and Hua (19)	2010.01–2014.01	COPD + AE + RF	74/80	/	CT + Pantoprazole 40 mg Q12H	CT	ade
Zu (20)	2018.01–2019.01	COPD + GRED	42/41	9.6 ± 2.5/ 8.8 ± 1.9	CT + Esomeprazole	CT	bc
Zan et al. (21)	2012.01–2012.06	COPD + AE + GRED	48/50	/	CT + Omeprazole 20 mg BID	CT	c
Xiao (22)	2019.01–2019.09	COPD + AE + RF	120/120	9.12 ± 2.07/ 9.22 ± 2.64	CT + Pantoprazole 40 mg Q12H	CT	be
Zhang et al. (23)	2015.1–2017.05	COPD + AE	102/50	/	CT + Pantoprazole	CT	abdef
Zhi et al. (24)	2017.11–2018.11	COPD + AE	60/30	13.1 ± 1.1	CT + Pantoprazole	CT	abde

*COPD, chronic obstructive pulmonary disease; AE, acute exacerbations; RF, respiratory failure; CT, conventional treatment; a, case fatality rate; b, clinical efficacy; c, Forced expiratory volume in one second/Forced vital capacity (FEV1/FVC); d, gastrointestinal bleeding; e, other adverse reactions; f, nosocomial infections; g, the number of acute exacerbations; /, unspecified*.

### Quality Assessment

**Figure 2:**
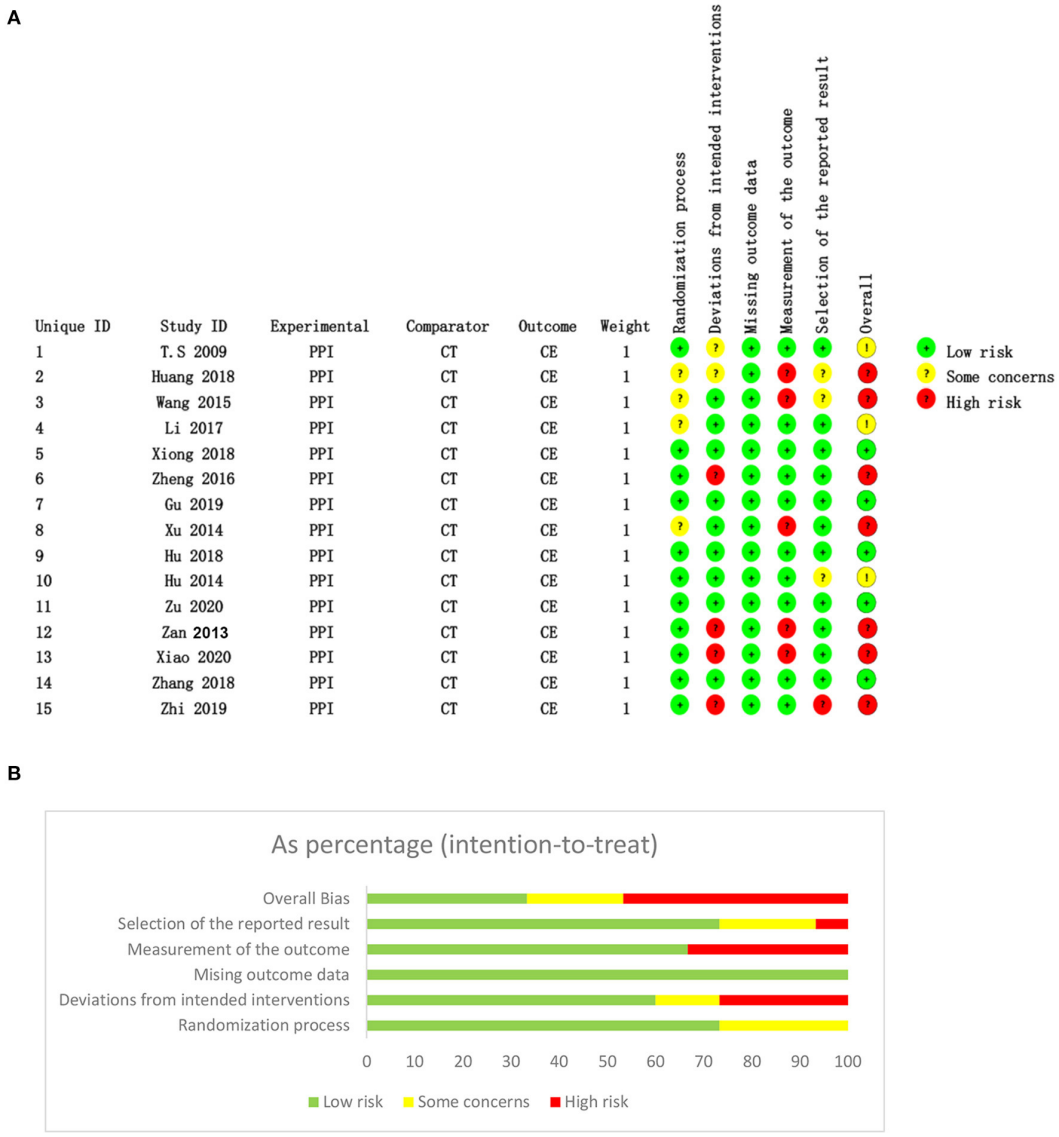
Risks of bias assessed by RoB2 for each included study (*n* = 15). **(A)** Risk of bias graph; **(B)** risk of bias summary. CT, conventional treatment; CE, clinical efficacy.

As previous mentioned, we used RoB2 to assess the risk of bias and the GRADE to rate the level of evidence of the outcomes in this meta-analysis (8, 9). Risk assessment results suggested that five studies were at low risk (14, 16, 18, 20, 23), three had some concerns (10, 13, 19) and seven studies had high risk (11, 12, 15, 17, 21, 22, 24). The risk of bias for each included study is shown in Figure 2 and the GRADE evidence levels of all outcomes are shown in Table 2.

**Table 2:** The evidence level of the outcomes obtained in this study was evaluated using GRADE.

**Quality assessment**							**No of patients**		**Effect**		**Quality**	**Importance**
**No of studies**	**Design**	**Risk of bias**	**Inconsistency**	**Indirectness**	**Imprecision**	**Other considerations**	**PPI**	**Conventional treatment**	**Relative (95% CI)**	**Absolute**		
**Case fatality rate**												
9	Randomized trials	Serious^a^	No serious inconsistency	No serious indirectness	No serious imprecision	Reporting bias^a^	16/523 (3.1%)	42/450 (9.3%)	RR 0.3 (0.18 to 0.52)	65 fewer per 1,000 (from 45 fewer to 77 fewer)	⊕⊕○○ LOW	
								8.6%		60 fewer per 1,000 (from 41 fewer to 71 fewer)		
**Clinical efficacy**												
5	Randomized trials	Serious^a^	No serious inconsistency	No serious indirectness	No serious imprecision	None	290/348 (83.3%)	213/266 (80.1%)	RR 1.1 (1.02 to 1.19)	80 more per 1,000 (from 16 more to 152 more)	⊕⊕⊕○ MODERATE	
								70%		70 more per 1,000 (from 14 more to 133 more)		
**Incidence of gastrointestinal bleeding**												
10	Randomized trials	Serious^a^	No serious inconsistency	No serious indirectness	No serious imprecision	Reporting bias^a^	16/584 (2.7%)	67/511 (13.1%)	RR 0.23 (0.14 to 0.38)	101 fewer per 1000 (from 81 fewer to 113 fewer)	⊕⊕○○ LOW	
								15.1%		116 fewer per 1,000 (from 94 fewer to 130 fewer)		
**Incidence of adverse reactions**												
11	Randomized trials	Serious^a^	No serious inconsistency	No serious indirectness	No serious imprecision	Reporting bias^a^	122/678 (18%)	334/635 (52.6%)	RR 0.33 (0.28 to 0.39)	352 fewer per 1000 (from 321 fewer to 379 fewer)	⊕⊕○○ LOW	
								48%		322 fewer per 1,000 (from 293 fewer to 346 fewer)		
**The number of acute exacerbations (Better indicated by lower values)**												
2	Randomized trials	Serious^a^	Serious^a^	No serious indirectness	No serious imprecision	None	113	113	–	MD 1.17 lower (1.75 to 0.6 lower)	⊕⊕○○ LOW	
**FEV1/FVC (Better indicated by lower values)**												
2	Randomized trials	Serious^a^	No serious inconsistency	Serious^a^	No serious imprecision	None	90	91	–	MD 3.94 higher (8.7 lower to 16.58 higher)	⊕⊕○○ LOW	
**Nosocomial infection rate**												
2	Randomized trials	Serious^a^	Serious^a^	No serious indirectness	Serious^a^	None	17/165 (10.3%)	7/113 (6.2%)	RR 1.31 (0.57 to 3)	19 more per 1,000 (from 27 fewer to 124 more)	⊕○○○ VERY LOW	
								6.8%		21 more per 1,000 (from 29 fewer to 136 more)		

a *means Risk of bias graph*.

### Case Fatality Rate

**Figure 3:**
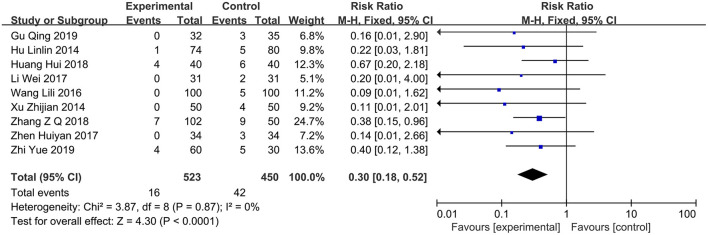
Forest plot of the case fatality rate between the PPI treatment group and the conventional treatment group. PPI, proton-pump inhibitor.

A total of nine studies (12, 13, 15–19, 23, 24) compared the case fatality rates between the PPI group (*n* = 523) and the control group (*n* = 450). The results of the heterogeneity test showed *I*^2^ = 0%, so the fixed-effects model was used for combined analysis. Results of the meta-analysis revealed that the case fatality rate of the PPI group can be significantly reduced compared with that of the control group (RR = 0.3; 95% CI, 0.18–0.52; *P* < 0.0001) (Figure 3).

### Clinical Efficacy

**Figure 4:**
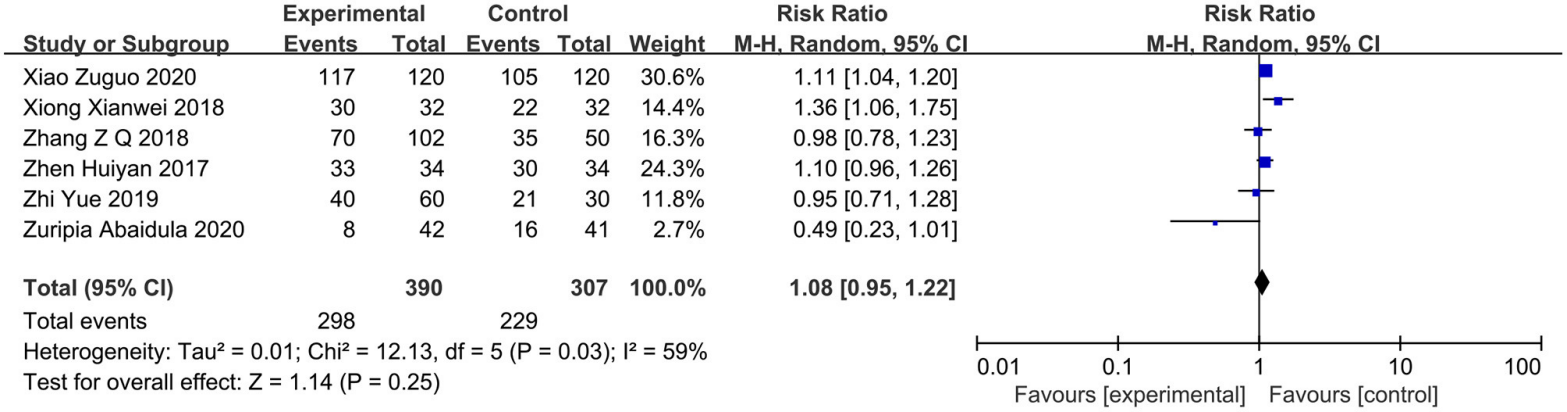
Forest plot of the clinical efficacy between the PPI treatment group and the conventional treatment group. PPI, proton-pump inhibitor.

Six studies (14, 15, 20, 22–24) reported the clinical efficacy of PPI therapy in the PPI group (*n* = 390) in comparison with the control group (*n* = 307). The heterogeneity test showed *I*^2^ = 59%. The sensitivity analysis suggested that the study conducted by Zu et al. (20) might be the source of heterogeneity. Therefore, a random-effects model analysis was used. The results of the meta-analysis presented the lack of significant difference between the two groups (RR = 1.08; 95% CI, 0.95–1.22; *P* = 0.25) (Figure 4).

### Incidence of Gastrointestinal Bleeding

**Figure 5:**
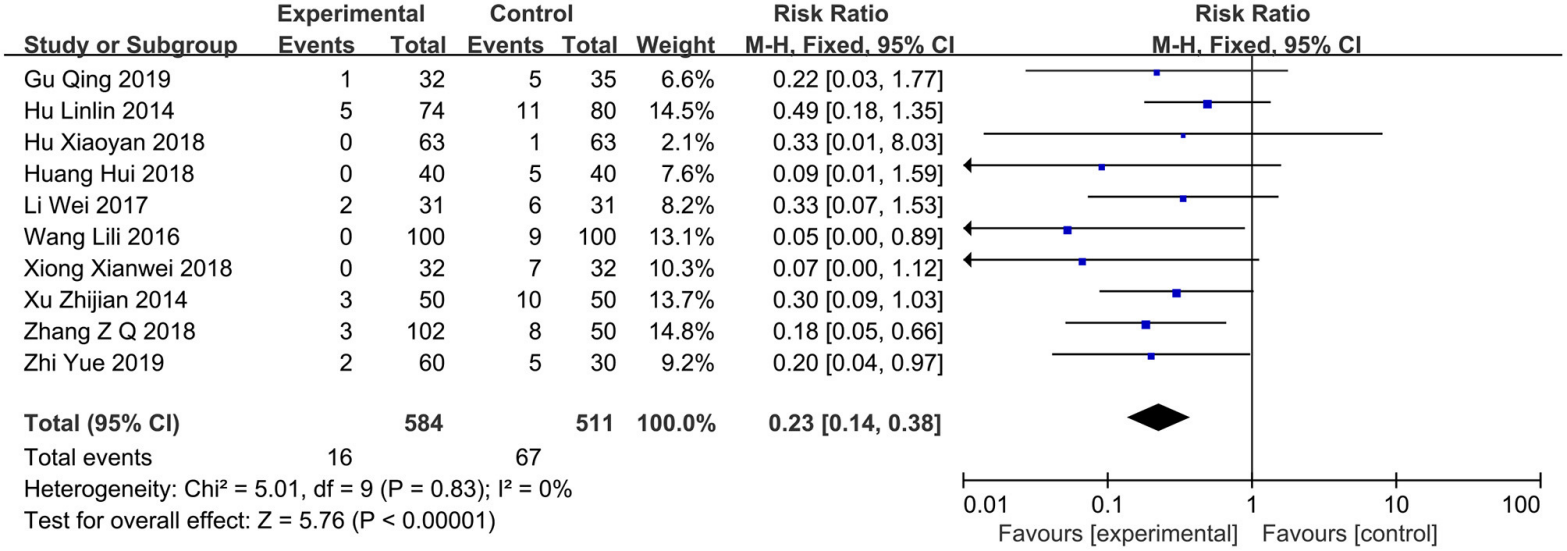
Forest plot of the incidence of gastrointestinal bleeding between the PPI treatment group and the conventional treatment group. PPI, proton-pump inhibitor.

A total of 10 studies (12–14, 16–19, 23, 24) reported gastrointestinal bleeding events, including 584 and 511 cases in the PPI group and control group, respectively. The heterogeneity test showed *I*^2^ = 0%; therefore, a fixed-effects model was employed. The results of the meta-analysis revealed that the incidence of gastrointestinal bleeding can be significantly reduced in patients with COPD who received PPI (RR = 0.23; 95% CI, 0.14–0.38; *P* < 0.00001) (Figure 5).

### Incidence of Other Adverse Reactions

**Figure 6:**
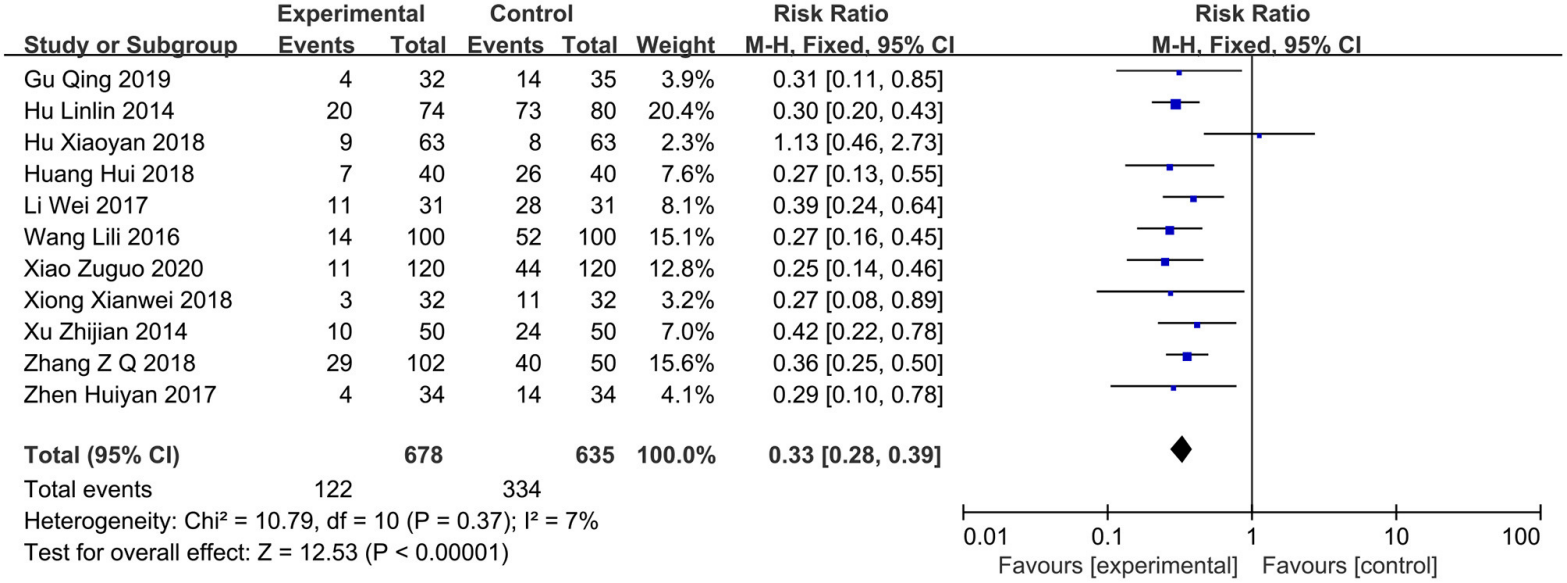
Forest plot of the incidence of other adverse reactions between the PPI treatment group and the conventional treatment group. PPI, proton-pump inhibitor.

Eleven studies (11–19, 22, 23) reported other adverse events except gastrointestinal bleeding, including 738 and 665 cases in the PPI group and control group, respectively. The heterogeneity test showed *I*^2^ = 0%; thus, a fixed-effects model was used. Compared with conventional treatment alone, PPI therapy in patients with COPD can reduce the incidence of other adverse reactions (RR = 0.33; 95% CI, 0.28–0.39; *P* < 0.00001) (Figure 6).

### Number of Acute Exacerbations

**Figure 7:**
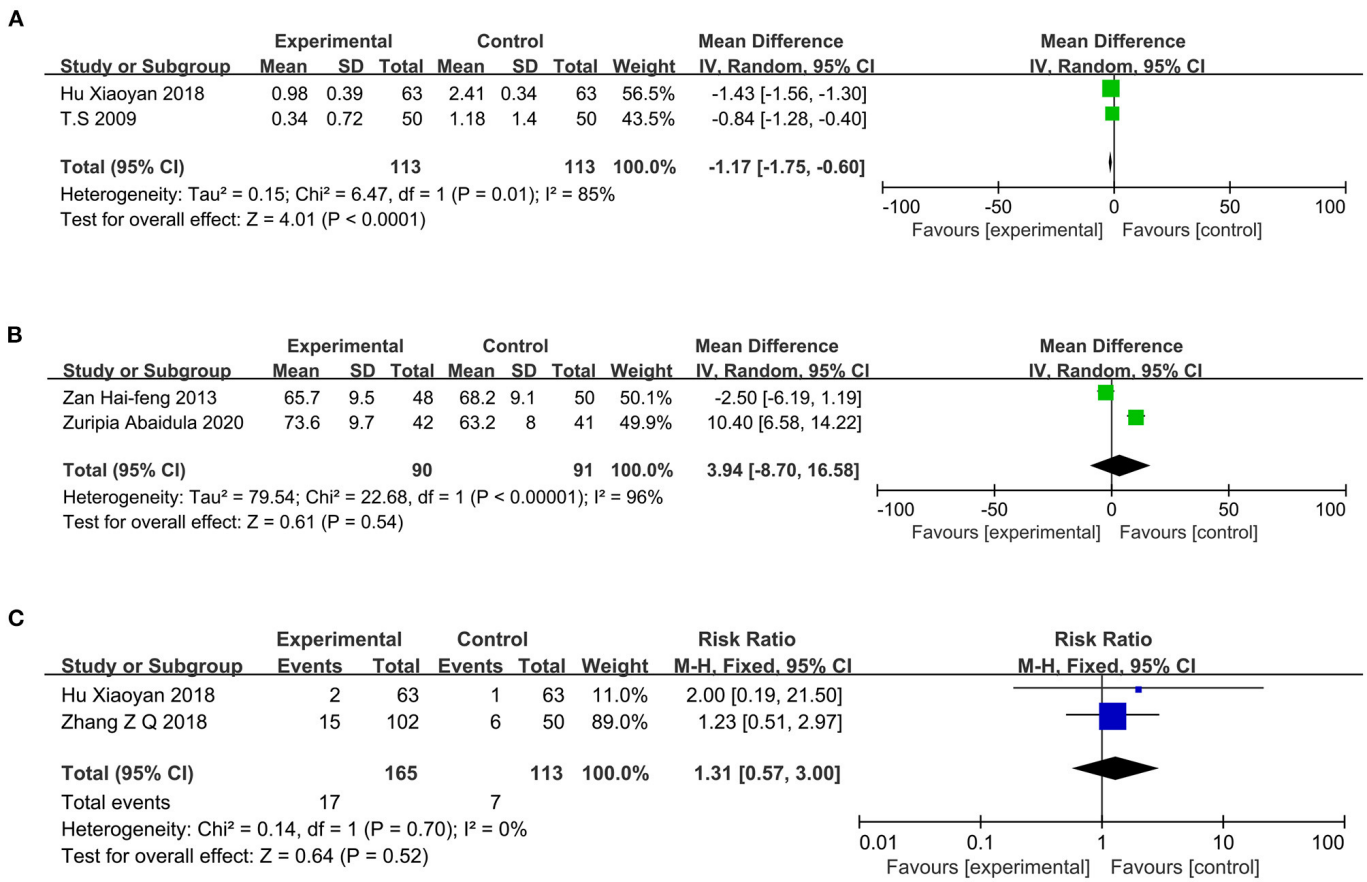
**(A)** Forest plot of the number of acute exacerbations between the PPI treatment and conventional treatment groups; **(B)** Forest plot of FEV1/FVC between the PPI treatment and conventional treatment groups; **(C)** Forest plot of nosocomial infection rate between the PPI treatment and conventional treatment groups. FEV1/FVC, forced expiratory volume in 1 s/forced vital capacity; PPI, proton-pump inhibitor.

Two studies (10, 18) reported the number of acute exacerbations, including 113 cases each in the PPI and control groups. The heterogeneity test showed *I*^2^ = 85%; therefore, a random-effects model was chosen. Compared with conventional treatment alone, PPI therapy in patients with COPD can reduce the number of acute exacerbations (MD = −1.17; 95% CI, −1.75 to −0.60; *P* < 0.0001) (Figure 7A).

### FEV1/FVC

Two studies (20, 21) reported on pulmonary ventilation function, including 90 and 91 cases in the PPI group and control group, respectively. The heterogeneity test showed *I*^2^ = 96%; therefore, a random-effects model was chosen. The results of the meta-analysis showed no significant difference between the two groups (MD = 3.94; 95% CI, −8.70 to −16.58; *P* = 0.54) (Figure 7B).

### Nosocomial Infection Rate

Two studies (18, 23) reported the occurrence of nosocomial infections, including 165 and 113 cases in the PPI group and control group, respectively. The heterogeneity test showed *I*^2^ = 0%; therefore, a fixed-effects model was used. The results of the meta-analysis revealed no significant difference between the two groups (RR = 1.31; 95% CI, 0.57–3.00; *P* = 0.52) (Figure 7C).

### Publication Bias

**Figure 8:**
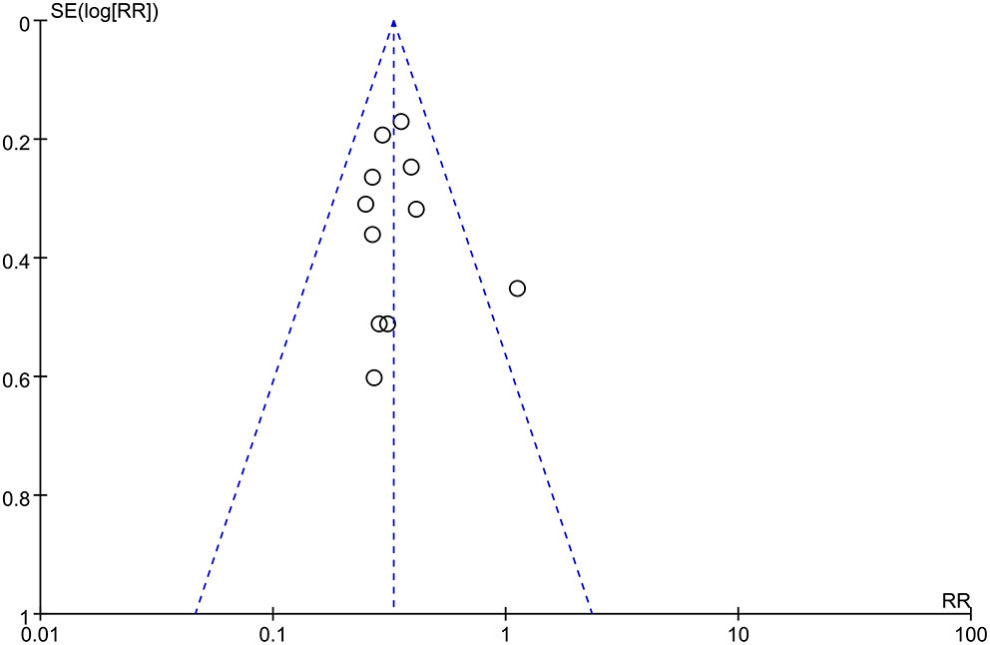
Funnel plot of the effect of proton-pump inhibitor therapy on patients with chronic obstructive pulmonary disease.

The present study included 15 studies, of which, 13 were from China. Therefore, there may be some publication bias in this study. We used funnel plots to verify publication bias (Figure 8). The graph that is not completely symmetrical indicated some publication bias. This is also one of the limitations of the present study.

## Discussion

Recently, several RCTs have focused on the role of PPI in patients with COPD. A study suggested that PPI therapy was safe and feasible in patients with COPD (25). A systematic review in the Cochrane database also tried to explore the effect of PPI therapy on patients with COPD. However, this study was only at the design stage, and no specific conclusions have been drawn (26). Therefore, we conducted this meta-analysis to clarify this issue. The results of this study suggested that PPI therapy in patients with COPD can reduce the case fatality rate, occurrence of gastrointestinal bleeding, other adverse reactions and number of acute exacerbations. The findings from this study may provide some guidance for the application of PPI in patients with COPD.

The results of the present study can be attributed to the following aspects: First, patients with COPD have long-term hypoxia, the gastrointestinal tract is the most sensitive organ for ischemia and hypoxia, and there are varying degrees of gastric mucosal damage. Especially, for older people who often have atherosclerosis and long-term use of non-steroidal drugs, the risk of gastrointestinal bleeding is high (27, 28). PPI is an H+/K+-ATPase inhibitor that has a strong inhibitory effect on gastric acid secretion and a protective effect on the gastric mucosa. It can effectively prevent and treat upper gastrointestinal bleeding, promote enteral nutrition support for patients immediately, enhance immunity and reduce abdominal distension and incidence of adverse reactions, such as diarrhea (25). Second, the clinical manifestations of COPD include repeated coughing, sputum expectoration and wheezing, which are closely related to a deteriorated condition (29). PPI can reduce the irritation of gastric acid and reflux of gastric contents on the esophagus and bronchi and relieve cough, sputum production and other uncomfortable clinical manifestations. Moreover, it can reduce the incidence of minor spiration caused by gastroesophageal reflux and avoid the occurrence of aspiration pneumonia (30). Third, previous studies have found that local or systemic inflammatory infection is an important factor for the pathogenesis of COPD, and more evidence supports the use of PPI to reduce inflammation (29–32). PPI can improve neurogenic inflammation, reduce plasma and sputum substance levels, block gastric acid secretion and selectively inhibited tumor necrosis factor-αand interleukin-1β secretion by Toll-like receptor-activated human monocytes *in vitro*, in the absence of toxic effects. Thus, the risk of infection in patients with COPD was reduced (1, 2). Fourth, mortality outcomes of patients with COPD are closely related to the frequency of acute exacerbations. PPI can reduce the risk of infection and the number of acute exacerbations in COPD patients, thereby reducing the risk of death. Fifth, 12 RCTs included in this study enrolled patients with acute exacerbations or even respiratory failure requiring hospitalization. Such patients have poor lung function on admission. Conventional treatments such as antibiotic therapy, nebulisation, resolving phlegm and administration of antispasmodic and anti-asthmatic drugs have contributed most to the improvement of respiratory function. Compared with conventional treatment, short-term PPI therapy during hospitalization cannot show a significant improvement in FEV1/FVC indicators. Sixth, in recent years, studies have found that the intestinal microbiota can regulate the systemic immune response, thereby affecting the function of extraintestinal organs. The gut-lung axis has received increasing attention on whether long-term PPI therapy causes bacterial overgrowth in the small intestine, bacterial peritonitis, intestinal flora shift, etc. There is currently no high-quality evidence (25, 33–35).

The preset study analyzed the effect of PPI therapy on the occurrence of nosocomial infections in patients with COPD, and tried to explore the contribution of PPI to the overall inflammatory response. However, two articles were finally included. The results were not statistically significant, and the effect of PPI therapy on the occurrence of nosocomial infections in patients with COPD has not been proved yet.

However, this study has the following limitations: First, the included studies had fewer patients with stable COPD, the study sample size was limited, and the clinical data of the population needed to be integrated to further improve the evidence. Second, most of the included studies did not report specific randomization methods and allocation concealment, and there was a greater risk of bias. Third, most of the included studies focus on Asian populations with limited geographic distribution; thus, multi-ethnic population studies are needed to provide evidence. Fourth, this meta-analysis had some publication bias. Finally, the high heterogeneity of some results may affect the reliability of these results, thus, we used a random-effects model to combine the results to make the results more reliable.

In summary, the currently available limited evidence shows that PPI therapy of patients with COPD can reduce the case fatality rate, incidence of adverse reactions including gastrointestinal bleeding and number of acute exacerbations. At present, PPI therapy is not yet recommended in COPD guidelines. PPI is mainly used in patients with digestive system diseases. Therefore, PPI therapy may be beneficial to patients with COPD with high-risk factors of the digestive system.

## Conclusion

PPI therapy has significant effects on patients with COPD in reducing the number of acute attacks, adverse reactions, and mortality. This conclusion requires further verification in larger-scale RCTs.

## Data Availability Statement

The original contributions presented in the study are included in the article/supplementary material, further inquiries can be directed to the corresponding author/s.

## Author Contributions

FY and QH: conceptualization, writing, review, and editing. FY, YY, and QH: methodology. LZ and QH: supervision. LZ: project administration. All authors contributed to the article and approved the submitted version.

## Funding

This study was funded by Hefei Key Discipline Construction Funding Project [No: Hefei Health Science Education (2019) No. 160] and Hefei Famous Medical Studio Funding Project [Hefei Talent (2019) No. 1].

## Conflict of Interest

The authors declare that the research was conducted in the absence of any commercial or financial relationships that could be construed as a potential conflict of interest.

## Publisher's Note

All claims expressed in this article are solely those of the authors and do not necessarily represent those of their affiliated organizations, or those of the publisher, the editors and the reviewers. Any product that may be evaluated in this article, or claim that may be made by its manufacturer, is not guaranteed or endorsed by the publisher.
